# Clinical Profile, of Adults Hospitalised with Mpox and Exploratory Associations with HIV Infection and Selected Comorbidities in South Kivu, Democratic Republic of the Congo: A Multicentre Retrospective Study

**DOI:** 10.3390/v18080845

**Published:** 2026-08-01

**Authors:** Christian Tshongo Muhindo, Isaac Barhishindi, Arsène Daniel Nyalundja, Marina Saleeb, Mudarshiru Bbuye, Bruce Kirenga, Misaki Wayengera, Espoir Bwenge Malembaka, David Lupande Mwenebitu, Samuel Makali, Susanne Krasemann, Mannix Masimango, Samir Kumar-Singh, Robert Colebunders, Patrick D. M. C. Katoto, Joseph Nelson Siewe Fodjo

**Affiliations:** 1 Center for Tropical Diseases and Global Health, and Faculty of Medicine, Catholic University of Bukavu, Bukavu P.O. Box 285, Democratic Republic of the Congo; barhishindi.kibalama@ucbukavu.ac.cd (I.B.); nyalu_arse.adn@outlook.com (A.D.N.); bwenge.malembaka@ucbukavu.ac.cd (E.B.M.); lupande2000@gmail.com (D.L.M.); makali.lwamushi@ucbukavu.ac.cd (S.M.); masimango.imani@ucbukavu.ac.cd (M.M.); katotopatrick@gmail.com (P.D.M.C.K.); 2 Global Health Institute, University of Antwerp, 2000 Antwerp, Belgium; marina.saleeb@uantwerpen.be (M.S.); robert.colebunders@uantwerpen.be (R.C.); josephnelson.siewefodjo@uantwerpen.be (J.N.S.F.); 3 Hôpital Provincial Général de Référence de Bukavu, Université Catholique de Bukavu, Bukavu P.O. Box 285, Democratic Republic of the Congo; 4 Makerere Lung Institute, Makerere University College of Health Sciences, Mulago Hospital, Kampala P.O. Box 7062, Uganda; mudarshirubbuye@gmail.com (M.B.); brucekirenga@yahoo.co.uk (B.K.); waymisaki@gmail.com (M.W.); 5 Institute of Neuropathology, University Medical Center Hamburg-Eppendorf, 20246 Hamburg, Germany; 6 Department of Molecular Morphology-Microscopy, Faculty of Medicine and Health Sciences, University of Antwerp, 2000 Antwerp, Belgium; samir.kumarsingh@uantwerpen.be; 7 Division of Epidemiology and Biostatistics, Cochrane South Africa, South African Medical Research Council, Cape Town 7505, South Africa; 8 Department of Global Health, Faculty of Medicine, Stellenbosch University, Stellenbosch 7600, South Africa

**Keywords:** mpox, HIV infection, coinfection, comorbidity, severity of illness index, hospitalisation, retrospective studies, adult, Democratic Republic of the Congo

## Abstract

Mpox remains endemic in the Democratic Republic of the Congo (DRC), where co-endemic infections and non-communicable comorbidities may influence disease severity and clinical outcomes. However, data on associated factors of adverse outcomes among hospitalised adults with mpox in African settings remain limited. This study aimed to describe the clinical profile of adults hospitalised with mpox in South Kivu Province, eastern DRC, and to explore associations between selected comorbid conditions, including HIV infection, malaria, and hyperglycaemia, and mpox disease severity, mortality, and hospitalisation duration. We conducted a multicentre retrospective observational study among adults admitted to five mpox treatment centres in South Kivu Province, DRC, between January 2024 and December 2025. Demographic, clinical, and laboratory data were extracted from routine hospital registers. Mpox disease severity (available only for a subset of participants) was classified according to WHO criteria. Participants included adults with suspected, probable or laboratory-confirmed mpox according to WHO case definitions in use during the study period. In addition to mpox disease severity, outcomes of interest included in-hospital mortality and duration of hospitalisation. Multivariable regression models were used to explore associations between selected comorbidities and clinical outcomes after adjustment for age and sex. Among 652 hospitalised adults included in the analysis, the median age was 26 years (IQR 21.0–35.0), and 383/652 (58.7%) were female. Among patients with recorded severity data (*n* = 104), moderate or severe mpox was documented in 88/104 (84.6%) patients. Overall mortality was 14/645 (2.2%). HIV infection was identified in 5 of the 71 participants tested (7.0%). Among participants with available test results, HIV infection appeared to be associated with higher mpox severity and mortality. Increasing age, but not HIV infection, malaria, or glycaemic status, was associated with longer hospitalisation. Because laboratory investigations were performed in only a subset of participants and missing data were substantial, these findings should be interpreted cautiously and considered exploratory. Prospective studies incorporating systematic laboratory testing and standardised clinical data collection are needed to better define factors associated with severe mpox in endemic African settings.

## 1. Introduction

Mpox, formerly referred to as monkeypox, remains endemic in the Democratic Republic of the Congo (DRC), where overlapping infections and comorbidities may influence disease severity and outcomes [1]. In 2022, mpox became a global health priority when the World Health Organisation (WHO) declared a Public Health Emergency of International Concern (PHEIC) for the multi-country outbreak by the subclade IIb (23 July 2022), which ended in May 2023; WHO declared a second PHEIC for mpox on 14 August 2024, following an upsurge in infections with the subclade Ib originating in the DRC and spreading to neighbouring countries [2]. Two major outbreaks have since been documented, in 2022 and 2024 [3]. The most recent outbreak, originating in South Kivu (DRC), remains active, although incidence is currently declining [4,5]. According to the WHO’s September 2025 report, the DRC accounted for approximately 38% of reported global cases and 17% of reported deaths [4].

This protracted outbreak underscores the risk of sustained community transmission [4] in a region already burdened by other endemic diseases, such as malaria [6]. Addressing this challenge requires the implementation of robust evidence to identify population groups at increased risk of severe disease and poor clinical outcomes, thereby supporting appropriate clinical management and public health interventions.

Previous studies have demonstrated that advanced or poorly controlled HIV infection, particularly in individuals with low CD4 cell counts or unsuppressed HIV viral load, is associated with an increased risk of severe mpox and death [7]. However, such studies remain limited, particularly in African populations [8]. Diabetes mellitus has also been consistently associated with increased susceptibility to viral infections [9], yet its role in mpox outcomes is poorly understood [10].

This study aims to describe the clinical characteristics of non-pregnant adults hospitalised with mpox in South Kivu. Specifically, to explore associations between HIV infection, malaria, admission hyperglycaemia and selected clinical outcomes, including mpox severity, duration of hospitalisation and in-hospital mortality.

## 2. Materials and Methods

### 2.1. Study Design

We conducted a retrospective study to describe the epidemiological and clinical characteristics of non-pregnant adults hospitalised with suspected, probable or confirmed mpox in South Kivu Province, DRC. We used routinely collected facility register data to provide a programmatic description of hospitalised cases admitted between January 2024 and December 2025.

#### Study Setting and Population

**Figure 1:**
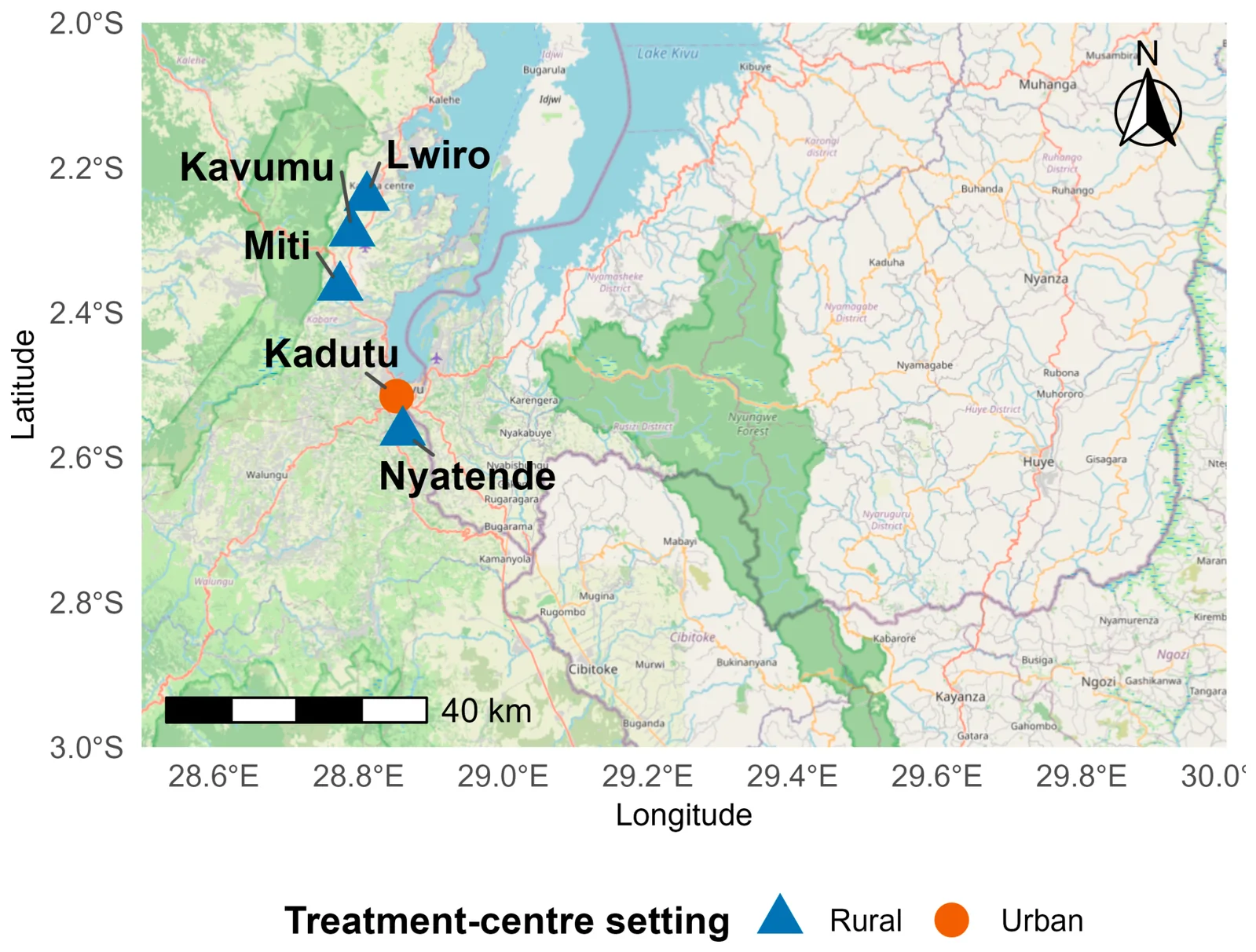
Location of the five participating mpox treatment centres in South Kivu Province, Democratic Republic of the Congo. The map was prepared using [R version 4.4.2 (R Foundation for Statistical Computing, Vienna, Austria) using basemap data from © OpenStreetMap contributors].

The study was conducted in five mpox treatment centres across South Kivu Province of DRC. One treatment centre (Kadutu) was located in an urban area in Bukavu town, which serves as a reference centre for the province, whereas four treatment centres were located in rural settings: Kavumu, Lwiro, Miti, and Nyatende (See Figure 1). Centres were selected based on high burden of mpox cases, geographical accessibility, and availability of patient records. Eligible participants were all non-pregnant adults admitted to the selected mpox treatment centres with suspected, probable or laboratory-confirmed mpox according to the WHO case definitions in use during the study period. In the hospitalisation registries, suspected and probable mpox were not distinguished. Laboratory confirmation by PCR was available for only a subset of participants because testing was not performed systematically during the outbreak. Pregnant women were excluded to maintain a homogeneous study population of non-pregnant adults. Because pregnancy is recognised as a risk factor for severe mpox and adverse maternal and foetal outcomes, the findings of this study cannot be generalised to pregnant women.

### 2.2. Data Collection

Patient information was extracted from routine treatment centre registers into Microsoft Excel spreadsheets using a standardised data extraction form. Collected variables included: sociodemographic characteristics (age and sex), anthropometric parameters (weight and height), clinical features (temperature, mpox WHO severity classification [11], biological parameters (mpox PCR, HIV rapid diagnostic test, random capillary glycaemia, thick smear, white blood cell count, neutrophil count, lymphocyte count, haemoglobin, platelet count), duration of hospitalisation and clinical outcome (discharged alive or died during hospitalisation). Mpox severity was classified according to the WHO clinical classification. Mild disease was defined as localised skin or mucosal lesions without systemic complications or need for inpatient supportive interventions. Moderate disease included more extensive skin or mucosal involvement or systemic symptoms requiring inpatient management but without life-threatening complications. Severe disease included widespread, sometimes confluent or necrotising lesions, severe mucosal involvement compromising nutrition or airway, major secondary bacterial infection, sepsis, encephalitis, ocular disease threatening vision, or other organ involvement requiring advanced supportive care.

Mpox diagnosis was confirmed using the GeneXpert^®^ Mpox assay (Cepheid, Sunnyvale, CA, USA; version M17.1), which detects clade I mpox virus. Specimens consisted of lesion swabs, lesion crusts or lesion roof material collected according to WHO recommendations. The GeneXpert platform provides qualitative results only; therefore, cycle threshold (Ct) values were not available.

### 2.3. Statistical Analysis

Extracted data were cleaned in Microsoft Excel 2021 (Microsoft Corp., Redmond, WA, USA) before being imported into R version 4.5.2 (R Foundation for Statistical Computing, Vienna, Austria; https://www.r-project.org/ (accessed on 27 May 2026)) for analysis. Categorical variables were presented as frequencies and percentages. Distribution of quantitative variables was assessed using the Shapiro–Wilk test (see Supplementary Materials, Table S1). As most continuous variables were not normally distributed, they were summarised using the median and interquartile range (IQR). Additionally, for some analyses the comorbidity variables were recoded into a dichotomous variable indicating the presence or absence of at least one comorbidity (HIV and/or hyperglycaemia and/or malaria). This pooling of comorbidities helped to increase the numbers for more robust statistical testing.

Comparisons between patients according to the outcomes were made using the two-tailed Pearson’s χ^2^ test (or Fisher’s exact test when at least one expected cell count was <5) for categorical variables. Continuous variables were compared using the Mann–Whitney U test when two independent groups were compared and the Kruskal–Wallis test when three or more independent groups were compared. An ordinal logistic regression model was used to identify factors associated with mpox severity (mild < moderate < severe). Gamma regression models with an identity link were used to identify associated factors of hospitalisation duration because it is a continuous, strictly positive, and skewed variable. All multivariable models were adjusted a priori for age and sex to account for potential confounding. Statistical significance was set at 5%.

Because several variables contained substantial missing data, analyses were performed using complete-case analyses for each statistical model. No multiple imputation was performed because the proportion of missing data was high and the missingness was considered unlikely to be completely at random. Consequently, the regression analyses should be regarded as exploratory and interpreted cautiously, as complete-case analyses may introduce selection bias if participants with complete laboratory data differ systematically from those without such data.

Given the retrospective nature of the study and the non-systematic availability of laboratory investigations, all regression analyses should be considered exploratory rather than confirmatory.

### 2.4. Ethical Considerations

This study was approved by the institutional Health Ethics Committee (Comité Institutionnel d’ Ethique de la Santé [CIES]) of the Université Catholique de Bukavu. Because this was a retrospective review of routinely collected anonymised hospital records, the requirement for individual informed consent was waived by the Ethics Committee. All procedures performed followed the ethical standards of the institutional ethical committee and the 1964 Declaration of Helsinki with its later amendments. The extracted data were anonymised such that no finding could be traced to a specific individual.

## 3. Results

### 3.1. Baseline Characteristics

Of 910 records reviewed, 33 were excluded due to pregnancy, and 225 because information on clinical outcome was unavailable. Ultimately, 652 (71.6%) patients with available sociodemographic information and partial/complete clinical data in the treatment registers were included in the study. The median age of the study population was 26.0 years (IQR: 21.0–35.0 years), with 58.7% of the participants being female. Malaria smears and mpox PCR were missing in nearly 70% of records. Weight, height, temperature and mpox severity were missing in approximately 80% of records. CRP, WBC, haemoglobin, platelets, HIV status, and glycaemia were missing in more than 90% of cases. The availability of laboratory investigations depended on routine clinical practice rather than a predefined study protocol. Consequently, laboratory testing was not systematic and missing data were likely not random. Age and sex variables were complete. Totals vary across variables due to missing data (Table 1).

### 3.2. Comparison of Characteristics by Mpox PCR Result

Among the subset of participants with PCR results (*n* = 177), we found no significant differences between those with PCR-positive results (confirmed mpox) and PCR-negative (suspected or probable mpox) (Table 2). However, because PCR testing was performed in only a subset of participants and the PCR-negative group was relatively small, these findings should not be interpreted as evidence of equivalence between the two groups. Because this study aimed to describe routine clinical practice during the outbreak, subsequent analyses included all participants meeting the WHO clinical case definitions for suspected, probable or confirmed mpox.

### 3.3. Mpox and Comorbidities

Concerning comorbidities, median random glycaemia was 92.0 mg/dL [IQR 84.0–104.0 mg/dL] and 3.8% of patients (2/53) had random glycaemia ≥ 200.0 mg/dL (hereafter referred to as hyperglycaemia). HIV infection was identified in 5 of the 71 participants tested (7.0%), while 8.0% (16/200) had a positive malaria thick smear. Hyperglycaemia (random capillary glucose ≥ 200 mg/dL) was observed in 2 of the 53 participants tested (3.8%).

#### Hospitalisation and Outcomes

The median length of hospital stay was 9.0 days [IQR 6.0–12.0 days], and 71.1% (86/121) of the participants were hospitalised for more than 7.0 days. Overall mortality of the hospitalised participants at the mpox treatment centres was 2.2% (14/645).

Table 3 compares participants according to the severity of mpox. Those with severe mpox (*n* = 20) were slightly older compared to those with mild (*n* = 16) and moderate (*n* = 68) forms, but this difference was not statistically significant (27.5 [22.0–33.2] years vs 26.5 [22.5–30.0] years and 26.0 [21.8–33.2] years, respectively; *p* = 0.872). Severe cases of mpox had a higher body temperature at admission (36.9 °C [36.5–38.0] compared to mild and moderate cases; *p* = 0.006). HIV status showed a significant association with mpox severity (*p* = 0.006): Persons with HIV constituted 30% of all participants with severe mpox for whom HIV serology was known (*n* = 10), much higher than the 14.3% of severe mpox cases diagnosed with hyperglycaemia. Thick smears were negative for all participants for whom mpox severity data were reported. Participants with severe mpox more frequently had documented HIV infection than those with mild or moderate disease. No consistent association between malaria or hyperglycaemia and disease severity was observed. Because laboratory investigations were available for only a subset of participants, these findings should be interpreted cautiously.

### 3.4. Associations with Severity and Mortality

Compared with survivors (*n* = 631), mpox participants who died (*n* = 14) were more frequently HIV positive (75.0% vs 3.0% HIV in survivors; *p* = 0.001), more often had at least one comorbidity, and more often had a longer hospitalisation duration (Table 4). All deaths among participants with available severity data occurred in individuals classified as having severe mpox.

Among participants who underwent HIV testing, HIV infection was more frequently observed among those who died during hospitalisation. However, only four deceased participants underwent HIV testing, and three tested positive. Therefore, this finding is based on very small numbers and should be interpreted cautiously.

### 3.5. Associations Between Mpox Severity/Outcomes and Presence of Comorbidities

In adjusted models, the presence of at least one comorbidity was associated with higher odds of greater mpox severity (aOR 20.7 [95% CI: 2.3–457.9]) and death (aOR 5.7 [1.3–24.6]) (Table 5). Confidence intervals were wide, consistent with small complete-case denominators.

The Gamma regression model presented in Table 6 investigated associated factors for increasing number of days of hospitalisation at the mpox treatment centre. Age emerged as the only predictor of prolonged hospitalisation, while HIV infection status and glycaemia were not significantly associated with hospitalisation duration. Of note, malaria (positive thick smear) was excluded from the gamma regression models because it presented only one level (negative) within the subgroup with complete data required for model estimation.

## 4. Discussion

This multicentre study conducted in South Kivu aimed to assess factors associated with adverse outcomes among non-pregnant adult participants hospitalised for mpox. The findings highlight significant challenges in data completeness, with up to 90% missingness for some variables. This limitation constrains inference and underscores the need for standardised admission forms and systematic clinical and laboratory data collection. The results indicate that most participants presented with moderate (65.4%) or severe (19.2%) forms of the disease, reflecting a substantial clinical burden, and an overall mortality of 2.2%. Age emerged as the sole predictor associated with prolonged hospitalisation, while HIV infection appeared to be associated with disease severity and mortality, although estimates were based on very small numbers of tested individuals and CD4 counts were not measured. In contrast, glycaemia levels did not differ significantly with mpox severity nor were they associated with increased fatalities, although elevated levels were noted among deceased participants. Only 53 participants had glycaemia recorded, and only two had hyperglycaemia ≥200 mg/dL, limiting statistical power. Given increasing diabetes prevalence and the known association between dysglycaemia and infection severity in other settings, systematic glycaemia measurement in hospitalised mpox patients remains justified. HIV testing was only available for 71 out of 652 patients, and glycaemia for 53 patients. It is highly likely that clinicians only ordered these tests for patients presenting with severe illness or specific risk factors. Malaria thick smear results showed no significant association with poor outcomes or mortality, and severity analysis was limited by incomplete data.

The mpox cases reported in South Kivu are related to clade Ib, a sublineage of clade I, which is known to be associated with severe clinical symptoms [5,12]. However, overall mortality was relatively low (2.2%). Although recent WHO reports indicate that the overall case fatality ratio associated with clade Ib has generally been below 1%, these estimates include many community-managed cases. Our study included only hospitalised adults and therefore represents a population with more severe illness, which likely explains the higher mortality observed. Previous studies have described the general epidemiology, clinical presentation, and outcomes of mpox in South Kivu and the broader DRC [5,13,14,15]. Our study specifically focused on the association of prevalent comorbidities such as malaria, HIV infection, and hyperglycaemia with mpox to assess their impact on the clinical presentation and mortality.

HIV co-infection was identified in 7.0% (5/71) of the participants tested, which is reportedly higher than the 2% reported by Brosius et al. in their study conducted at the General Hospital of Kamituga, the epicentre of the 2024 outbreak, between May and October 2024 [14]. This discrepancy may reflect bias and under-reporting due to reliance on self-reported data. Cibenda R. et al. reported a lower prevalence of HIV positivity (2.6%) among mpox patients, from July to December 2024, likely due to inclusion of participants without confirmatory mpox test results. In contrast, records from the South Kivu provincial surveillance authorities from September 2023 to February 2024, reported by Vakaniaki E. et al. [12], indicate a prevalence of 6.5% (3/46), aligning more closely with our findings. Previous studies have shown that severe mpox occurs predominantly among individuals with advanced or poorly controlled HIV infection, particularly those with low CD4 cell counts or unsuppressed HIV viral load. Unfortunately, CD4 cell counts and HIV viral load measurements were unavailable in our study, preventing further assessment of the degree of immunosuppression. Taken together, these findings emphasise the importance of recognising HIV co-infection in mpox patients, particularly among individuals with advanced poorly controlled HIV infection. In our study, HIV infection appeared to be associated with disease severity and mortality; however, HIV testing was performed in only a small subset of participants and was most likely requested preferentially in those with severe illness. Consequently, these findings should be interpreted cautiously because testing-by-indication bias cannot be excluded.

In our study, glycaemia did not differ significantly across severity or outcome groups. A previous study conducted during the COVID-19 pandemic in South Kivu, at the Hôpital Provincial Général de Référence de Bukavu, showed that hyperglycaemia was independently associated with hospital mortality [9]. High glucose levels have been shown to impair DNA sensors (such as TLR9 and cGAS), which suggests that diabetic patients could be exceptionally susceptible to DNA viruses like the mpox virus (10). However, a single random capillary glucose measurement obtained during an acute febrile illness cannot distinguish pre-existing diabetes mellitus from transient stress hyperglycaemia, and this should be considered when interpreting our findings. The lack of statistical significance in our study may be explained by the small sample size of participants with glycaemia results (53/652), of whom only two (3.8%) had random glycaemia levels above 200 mg/dL, which is the threshold used in the COVID-19 study to define hyperglycaemia at admission. Poor glucose control is recognised as a risk factor for susceptibility and severity of infections [10,16,17], and given the rising prevalence of diabetes in South Kivu [17], monitoring glycaemia in patients hospitalised for mpox is warranted. Further studies are necessary to clarify this association and its implications for patient management.

In our study, the prevalence of positive malaria thick smear was 8%, which is consistent with the 7% reported by Cibenda R. et al. [15]. We found no significant association with mortality, and severity analysis was limited by incomplete data. Similarly, admission hyperglycaemia showed no consistent association with disease severity, mortality or duration of hospitalisation, although these analyses were based on very small numbers of participants with available laboratory results. While co-infections and metabolic parameters may play a role in the clinical outcomes of mpox patients, they may be secondary to the effects of immunological status, particularly in patients with HIV. Identifying population groups that are particularly vulnerable, such as those requiring prioritised vaccination access, is essential for effective public health interventions.

This study has important limitations. The retrospective design resulted in substantial missing data and heterogeneity in data collection from different registers by various research assistants without standardised reporting. Because of the retrospective observational design, causal inferences cannot be established. Furthermore, laboratory investigations, including HIV testing, glycaemia measurements and malaria thick smears, were not performed systematically but according to routine clinical judgement. Consequently, testing-by-indication bias may have influenced the observed associations between these comorbidities and adverse clinical outcomes. Additionally, the inability to disentangle suspected and probable mpox cases owing to the limited mpox PCR testing among participants poses a challenge. Moreover, only a subset of participants underwent laboratory confirmation, and inclusion of clinically diagnosed suspected and probable cases may have resulted in disease misclassification. Although no statistically significant differences were observed between PCR-positive and PCR-negative participants with available laboratory results, this should not be interpreted as evidence of equivalence because PCR testing was available for only a subset of participants. Also, the small complete-case denominators for multivariable models produced imprecise estimates, reflected in wide confidence intervals. Consequently, participants included in the regression analyses may not be representative of the overall study population. The relatively low mortality observed may reflect under-ascertainment of deaths, referral patterns, survivor bias, or inclusion of clinically suspected cases without virological confirmation. Finally, CD4 cell counts and HIV viral load measurements were unavailable, preventing assessment of the degree of immunosuppression among participants living with HIV, and pregnant women were excluded; therefore, our findings cannot be generalised to this high-risk population.

## 5. Conclusions

In this retrospective multicentre study in South Kivu among hospitalised adults with mpox, HIV infection appeared to be associated with severe disease and mortality, whereas malaria and admission hyperglycaemia showed no consistent association with adverse outcomes. However, interpretation is limited by extensive missing data, incomplete laboratory confirmation, non-systematic laboratory testing, possible testing-by-indication bias, and small analytical sample sizes. Therefore, these findings should be regarded as exploratory. Prospective studies with systematic laboratory testing, including assessment of CD4 cell counts and HIV viral load among participants living with HIV, and standardised clinical data collection are needed to better define factors associated with severe mpox in endemic African settings.

## Figures and Tables

**Table 1:** Characteristics of 652 study participants.

	Findings	Totals (N) *
Age in years: Median (IQR)	26.0 [21.0–35.0]	652
Age groups: *n* (%)		
18–24 years	294 (45.1%)	
25–39 years	249 (38.2%)	
40–54 years	70 (10.7%)	
55–69 years	31 (4.8%)	
≥70 years	8 (1.2%)	
Sex: Female: *n* (%)	383 (58.7%)	652
BMI (Kg/m^2^): Median (IQR)	24.0 [21.5–26.0]	120
Temperature (°C): Median (IQR)	36.5 [36.0–37.0]	112
Mpox severity: *n* (%)		104
Mild	16 (15.4%)	
Moderate	68 (65.4%)	
Severe	20 (19.2%)	
Positive PCR mpox: *n* (%)	143 (80.8%)	177
CRP (mg/L): Median (IQR)	3.0 [3.0–12.8]	61
WBC (×10^3^ cells/μL): Median (IQR)	5.1 [3.5–7.3]	63
Neutrophils (×10^3^ cells/μL): Median (IQR)	4.2 [2.3–5.3]	21
Lymphocytes (×10^3^ cells/μL): Median (IQR)	1.6 [1.2–2.4]	61
Haemoglobin (g/dL): Median (IQR)	14.1 [12.9–15.7]	63
Platelets (×10^3^ cells/μL): Median (IQR)	197 [159.0–258.0]	61
Positive Malaria smear: *n* (%)	16 (8.0%)	200
Positive HIV test: *n* (%)	5 (7.0%)	71
Glycaemia (mg/dL): Median (IQR)	92.0 [84.0–104.0]	53
Glycaemia ≥ 200 mg/dL: *n* (%)	2 (3.8%)	53
Presence of at least one comorbidity **: *n* (%)	22 (8.9%)	247
Death: *n* (%)	14 (2.2%)	645
Duration of hospitalisation (days): Median (IQR)	9.0 [6.0–12.0]	121
More than 7 days hospitalisation: *n* (%)	86 (71.1%)	121

* Total number of participants with available information for the variable; ** defined as HIV infection (5/71), hyperglycaemia (≥200 mg/dL) (2/53), and/or malaria positivity (16/200); abbreviations: BMI, body mass index; CRP, C-reactive protein; HIV, Human Immunodeficiency Virus; IQR, interquartile range; PCR, polymerase chain reaction; WBC, white blood cell.

**Table 2:** Comparison of PCR-positive and PCR-negative participants hospitalised in mpox treatment centres.

	Positive *N* = 143	Negative *N* = 34	*p*-Value
Age in years: Median (IQR)	28.0 [21.0–35.0]	23.0 [20.0–30.0]	0.077
Sex: Female: *n* (%)	65/143 (45.5%)	22/34 (64.7%)	0.068
BMI (Kg/m^2^): Median (IQR)	24.1 [22.6–26.9]	23.9 [21.2–25.1]	0.465
Temperature (°C): Median (IQR)	36.7 [36.1–37.3]	36.7 [36.7–37.0]	0.633
Mpox severity: *n* (%)			1.000
Mild	3/31 (9.7%)	0/3 (0.0%)	
Moderate	18/31 (58.1%)	2/3 (66.7%)	
Severe	10/31 (32.3%)	1/3 (33.3%)	
CRP (mg/L): Median (IQR)	3.0 [3.0–12.0]	17.6 [10.3–24.9]	0.605
WBC (×10^3^ cells/μL): Median (IQR)	5.5 [3.5–8.5]	7.6 [6.2–9.1]	0.585
Neutrophils (×10^3^ cells/μL): Median (IQR)	5.2 [3.2–6.1]	4.9 [3.9–6.0]	1.000
Lymphocytes (×10^3^ cells/μL): Median (IQR)	1.6 [1.2–2.0]	1.9 [1.5–2.2]	0.775
Haemoglobin (g/dL): Median (IQR)	14.3 [12.7–15.6]	13.1 [12.9–13.3]	0.445
Platelets (×10^3^ cells/μL): Median (IQR)	178 [154.0–224.0]	233 [212.0–254.0]	0.402
Positive Malaria smear: *n* (%)	4/44 (9.1%)	0/12 (0.0%)	0.567
Positive HIV test: *n* (%)	3/25 (12.0%)	0/3 (0.0%)	1.000
Glycaemia(mg/dL): Median (IQR)	91.5 [83.2–100.0]	89.0 [89.0–89.0]	0.741
Glycaemia ≥ 200 mg/dL: *n* (%)	1/20 (5.0%)	0/1 (0.0%)	1.000
Presence of at least one comorbidity: *n* (%)	7/56 (12.5%)	0/15 (0.0%)	0.332
Duration of hospitalisation (days): Median (IQR)	12.0 [9.8–14.0]	10.5 [8.8–12.2]	0.622
More than 7 days hospitalisation: *n* (%)	34/36 (94.4%)	2/2 (100.0%)	1.000

**Table 3:** Comparison of findings by mpox severity.

	Mild (*N* = 16)	Moderate (*N* = 68)	Severe (*N* = 20)	*p*-Value *
Age (years): Median (IQR)	26.5 [22.5–30.0]	26.0 [21.8–33.2]	27.5 [22.0–33.2]	0.872
Sex: Female: *n* (%)	10/16 (62.5%)	26/68 (38.2%)	10/20 (50.0%)	0.180
BMI (Kg/m^2^): Median (IQR)	24.8 [24.4–26.0]	24.1 [22.7–26.2]	21.7 [19.9–24.6]	0.084
Temperature (°C): Median (IQR)	36.0 [36.0–36.4]	36.0 [36.0–37.0]	36.9 [36.5–38.0]	0.006
Positive PCR mpox: *n* (%)	3/3 (100.0%)	18/20 (90.0%)	10/11 (90.9%)	1.000
CRP (mg/L): Median (IQR)	3.5 [3.0–11.8]	3.0 [3.0–12.3]	3.0 [3.0–9.4]	0.659
WBC (×10^3^ cells/μL): Median (IQR)	4.9 [3.5–5.8]	5.0 [3.7–7.7]	4.4 [3.4–5.6]	0.668
Neutrophils (×10^3^ cells/μL): Median (IQR)	5.1 [3.3–5.2]	4.3 [2.9–5.7]	2.8 [1.5–3.6]	0.670
Lymphocytes (×10^3^ cells/μL): Median (IQR)	1.4 [1.0–1.6]	1.9 [1.4–2.5]	1.4 [1.0–2.1]	0.121
Haemoglobin (g/dL): Median (IQR)	13.0 [12.5–15.2]	14.2 [13.3–15.7]	13.8 [12.7–15.7]	0.587
Platelets (×10^3^ cells/μL): Median (IQR)	216 [152.0–279.0]	196 [167.0–244.0]	198 [174.0–276.0]	0.985
Positive Malaria smear: *n* (%)	0	0	0	
Positive HIV test: *n* (%)	0/9(0%)	0/40 (0%)	3/10 (30.0%)	0.006
Glycaemia(mg/dL): Median (IQR)	91.5 [83.5–97.2]	93.0 [86.0–104.0]	98.0 [88.5–106.0]	0.795
Glycaemia ≥ 200 mg/dL: *n* (%)	0/6 (0%)	1/33 (3.0%)	1/7 (14.3%)	0.490
Presence of at least one comorbidity: *n* (%)	0/9 (0%)	1/44 (2.3%)	3/11 (27.3%)	0.025
Death:	0/16 (0%)	0/67 (0%)	2/19 (10.5%)	0.056
Duration of hospitalisation (days): Median (IQR)	11.0 [7.5–13.0]	9.0 [6.0–12.8]	10.0 [8.0–13.0]	0.536
More than 7 days hospitalisation: *n* (%)	8/11 (72.7%)	30/42 (71.4%)	15/18 (83.3%)	0.690

Abbreviations: BMI, body mass index; CRP, C-reactive protein; HIV, Human Immunodeficiency Virus; PCR, polymerase chain reaction; WBC, white blood cell. * *p*-values are based on the Kruskal–Wallis test for continuous variables and Chi-squared tests or Fisher’s exact test for categorical variables.

**Table 4:** Comparison of findings between mpox participants with a fatal outcome and survivors.

	Survivors (*N* = 631)	Dead (*N* = 14)	*p*-Value *
Age (years): Median (IQR)	25.0 [21.0–35.0]	30.0 [24.2–36.2]	0.184
Sex: Female: *n* (%)	374/631 (59.3%)	8/14 (57.1%)	1.000
BMI (Kg/m^2^): Median (IQR)	24.0 [21.5–25.9]	23.3 [22.9–23.7]	0.673
Temperature (°C): Median (IQR)	36.5 [36.0–37.0]	37.9 [37.4–38.3]	0.081
Mpox severity: *n* (%)			0.056
Mild	16/100 (16.0%)	0/2 (0%)	
Moderate	67/100 (67.0%)	0/2 (0%)	
Severe	17/100 (17.0%)	2/2 (100.0%)	
Positive PCR mpox: *n* (%)	132/164 (80.5%)	6/8 (75.0%)	0.658
CRP (mg/L): Median (IQR)	3.0 [3.0–12.8]	8.0 [5.3–32.2]	0.344
WBC (×10^3^ cells/μL): Median (IQR)	4.8 [3.5–6.7]	9.1 [4.8–15.3]	0.336
Neutrophils (×10^3^ cells/μL): Median (IQR)	3.8 [2.3–5.2]	6.9 [4.0–9.8]	0.801
Lymphocytes (×10^3^ cells/μL): Median (IQR)	1.6 [1.2–2.3]	1.9 [0.8–4.2]	0.880
Haemoglobin (g/dL): Median (IQR)	14.3 [12.9–15.7]	12.0 [10.1–14.3]	0.175
Platelets (×10^3^ cells/μL): Median (IQR)	190 [156.0–250.0]	238 [153.0–308.0]	0.684
Positive Malaria smear: *n* (%)	16/192 (8.3%)	0/7 (0.0%)	1.000
Positive HIV test: *n* (%)	2/67 (3.0%)	3/4 (75.0%)	0.001
Glycaemia (mg/dL): Median (IQR)	91.5 [84.2–103.0]	139.0 [106.0–207.0]	0.336
Glycaemia ≥ 200 mg/dL: *n* (%)	1/50 (2.0%)	1/3 (33.3%)	0.111
Presence of at least one comorbidity: *n* (%)	19/237 (8.0%)	3/9 (33.3%)	0.037
Duration of hospitalisation (days): Median (IQR)	9.0 [6.0–12.0]	20.5 [15.8–23.2]	0.014
More than 7 days hospitalisation: *n* (%)	76/110 (69.1%)	4/4 (100.0%)	0.316

Abbreviations: BMI, body mass index; CRP, C-reactive protein; HIV, Human Immunodeficiency Virus; PCR, polymerase chain reaction; WBC, white blood cell. * *p*-values are based on the Kruskal–Wallis test for continuous variables and Chi-squared tests or Fisher’s exact test for categorical variables.

**Table 5:** Regression models investigating associations between mpox severity/fatal outcomes and presence of comorbidities.

Variable	OR	CI 95%	*p*-Value
Model 1: Ordinal logistic regression (all comorbidities combined), adjusted for age and sex			
Outcome: Mpox severity (mild = 0 vs. moderate = 1 vs. severe = 2); *n* = 64			
Age	1.0	[1.0–1.1]	0.507
Sex (Male)	1.6	[0.5–5.1]	0.436
Comorbidity present	20.7	[2.3–457.9]	0.014
Model 2: Classical logistic regression (comorbidities combined), adjusted for age and sex			
Outcome: Death (Yes vs no); *n* = 246			
Age	1.0	[1.0–1.1]	0.600
Sex (Male)	1.0	[0.3–4.1]	0.961
Comorbidity present	5.7	[1.3–24.6]	0.021

**Table 6:** Gamma regression models investigating factors associated with hospitalisation duration among participants.

Models	Estimation ß	CI 95%	*p*-Value
Model 1: Outcome: Number of days of hospitalisation; Comorbidity: HIV, adjusted for age and sex (*n* = 52)			
Age	0.2	[0.0–0.3]	0.013
Sex (Male)	−1.6	[−4.1–1.0]	0.231
HIV positive	2.9	[−2.3–8.2]	0.280
Model 2: Outcome: Number of days of hospitalisation; Comorbidity: Glycaemia as a continuous variable, adjusted for age and sex (*n* = 43)			
Age	0.2	[0.1–0.4]	0.004
Sex (Male)	−1.8	[−4.6–1.0]	0.212
Glycaemia (continuous)	−0.0	[−0.0–0.0]	0.113
Model 3: Outcome: Number of days of hospitalisation; Comorbidity: Hyperglycaemia (Glycaemia ≥ 200), adjusted for age and sex); (*n* = 43)			
Age	0.2	[0.1–0.4]	0.005
Sex (Male)	−1.7	[−4.6–1.1]	0.236
Hyperglycaemia	−4.0	[−8.4–0.3]	0.078
Model 4: Outcome: Number of days of hospitalisation; Comorbidity: Presence of any comorbidity, adjusted for age and sex (*n* = 54)			
Age	0.2	[0.1–0.3]	0.008
Sex (Male)	−2.1	[−4.5–0.4]	0.103
Comorbidity present	1.1	[−3.2–5.4]	0.616

## Data Availability

No new data were created as all data were extracted from the centre’s registry. For access to the dataset used for analysis, please contact the corresponding author.

## Supplementary Materials

The following supporting information can be downloaded at: https://www.mdpi.com/article/10.3390/v18080845/s1, Table S1: Shapiro-Wilk normality test.

## Abbreviations

The following abbreviations are used in this manuscript:

BMI	Body Mass Index
CI	Confidence Interval
CIES	Comité Institutionnel d’ Ethique de la Santé
CRP	C-reactive protein
DRC	Democratic Republic of the Congo
HIV	Human Immunodeficiency Virus
IQR	Interquartile Range
OR	Odds Ratio
PCR	Polymerase Chain Reaction
PHEIC	Public Health Emergency of International Concern
WBC	White Blood Cell
WHO	World Health Organisation
