# Supplementary material for: Clinical Profile, of Adults Hospitalised with Mpox and Exploratory Associations with HIV Infection and Selected Comorbidities in South Kivu, Democratic Republic of the Congo: A Multicentre Retrospective Study
Source: Viruses. 2026 Aug 1;18(8):845. doi: 10.3390/v18080845 (PMC13517878; doi:10.3390/v18080845)
Supplement: Supplementary file 1 [file viruses-18-00845-s001.zip › viruses-4375585-supplementary.pdf]

Shapiro-Wilk normality test

**Variables**	W	p-value
**Age**	0.82665	< 2.2e-16
**BMI**	0.95081	0.000248
**Temperature**	0.85898	6.307e-09
**CRP**	0.38252	1.381e-14
**WBC**	0.83761	8.254e-07
**Neutrophil**	0.88701	0.01975
**Lymphocyt**	0.8239	4.739e-07
**Hb**	0.98842	0.8202
**Platelets**	0.9168	0.0005111
**Glycaemia**	0.53585	1.068e-11
**Days_hospit**	0.93934	3.591e-05
